# Emotional intelligence and academic performance of medical undergraduates: a cross-sectional study in a selected university in Sri Lanka

**DOI:** 10.1186/s12909-017-1018-9

**Published:** 2017-09-25

**Authors:** Chandrani Nirmala Wijekoon, Heshan Amaratunge, Yashica de Silva, Solith Senanayake, Pradeepa Jayawardane, Upul Senarath

**Affiliations:** 1 Department of Pharmacology, Faculty of Medical Sciences, University of Sri Jayewardenepura, Gangodawila, Nugegoda, Sri Lanka; 2 Department of Community Medicine, Faculty of Medicine, University of Colombo, Colombo, Sri Lanka

**Keywords:** Emotional intelligence, Academic performance, Medical undergraduates, Sri Lanka

## Abstract

**Background:**

Emotional intelligence (EI) has been linked with academic and professional success. Such data are scarce in Sri Lanka. This study was conducted to describe the pattern of EI, to determine its predictors and to determine the effect of EI on academic performance at the final MBBS examination, in medical undergraduates of a Sri Lankan university.

**Methods:**

This is a cross-sectional study in a selected university, involving those who did final MBBS examination in 2016. Consecutive sampling was done. EI was assessed with self-administered Genos Emotional Intelligence Full Version (7 domains; 70 questions equally weighted; total score 350). Socio-demographic data were obtained using a self-administered questionnaire. Academic performance was assessed with final MBBS results in the first attempt.

**Results:**

Of 148 eligible students 130 responded (response rate-88%); 61.5% were females; mean age was 26.3 ± 1 years. Mean total EI score was 241.5 (females-245.5, males-235.1; *p* = 0.045).Among different domains, mean score was highest for Emotional Self-Awareness (36.8/50) and lowest for Emotional Expression (32.6/50). Multiple linear regression analysis indicated that having good family support (*p* = 0.002), socializing well in university (*p* = 0.024) and being satisfied with facilities available for learning (*p* = 0.002), were independent predictors of EI. At the final MBBS examination 51.6% obtained classes, 31.5% passed the examination without classes and 16.9% got repeated. Females had better academic performance than males (*p* = 0.009). Mean EI of second-class upper division, second-class lower division, pass and repeat groups were 249.4, 246.6, 240.2 and 226.9, respectively (with one-way ANOVA *p* = 0.015). After adjusting for gender, ordinal regression analysis indicated that, total EI score was an independent predictor of final MBBS results [β-0.018 (95% CI 0.005-0.031); *p* = 0.006].

**Conclusions:**

In the study population, both EI and academic performance were higher among females. Independent of gender, academic performance was better in those who were more emotionally intelligent. Several psychosocial factors were found to be independent predictors of EI. These results suggest that emotional skills development might enhance academic performance of medical undergraduates in Sri Lanka. Further research is needed in this under-explored area.

**Electronic supplementary material:**

The online version of this article (10.1186/s12909-017-1018-9) contains supplementary material, which is available to authorized users.

## Background

Emotional intelligence (EI) is defined as the ability to monitor one’s own and other people’s emotions, to discriminate between different emotions and label them appropriately, and to use emotional information to guide thinking and behaviour and to manage and/or adjust emotions to adapt to environments or achieve one’s goals [1]. Emotional intelligence comprises interpersonal and intrapersonal intelligence. Interpersonal intelligence is the outer intelligence one uses to understand and manage relationships with the other people. This is important for developing qualities like empathy and building up effective relationships. Intrapersonal intelligence is the inner intelligence one uses to know and understand oneself which is important for self-awareness, self-regulation and self-motivation. It can be postulated that management of interpersonal and intrapersonal emotions is vital for an individual’s academic and professional success. Those with higher EI are more likely to understand, regulate and manage emotions better both in themselves and in the others.

The concept of EI was first described by Salovey and Mayer more than two decades ago [2]. Subsequently, they described a four-branched model of EI [3]. The four branches or abilities were perceiving, using, understanding, and managing emotions. These different dimensions of EI are likely to influence the academic and professional success particularly in the field of medicine. The medical students learn and doctors work in a high-stress environment created by a multitude of factors which include demanding workloads, long hours of working, and having to interact with different personnel ranging from patients to healthcare teams. High level of stress and psychological distress among medical students and doctors are well documented [4, 5]. Studies have shown that higher EI is associated with lower levels of stress and better coping ability among medical students and other healthcare workers [6–8]. The different dimensions of EI help an individual to function effectively in a stressful environment. The first dimension of EI described by Salovey et al. [3] is, perceiving one’s own and others’ emotions. This is the first step in generating an appropriate response to stressful situations. The second dimension is, using emotions effectively to facilitate higher functions such as problem solving. It is a useful trait for a medical student to gain maximum use from the undergraduate training process and also to perform better at the examinations. Understanding emotions is the third dimension. This dimension includes the ability to be sensitive to own emotions and emotions of the others. This is an attribute that inculcates empathy which in turn helps to build up better interpersonal relationships. Having good interpersonal relationships with patients as well as with the ward staff is crucial for medical students to gain the maximum out of their clinical training. The fourth dimension is managing emotions in self and in the others. Managing ones emotions is extremely important to maintain psychological well-being during the stressful undergraduate years. Long term psychological well-being as well as psychological status during the examinations is likely to affect an individual’s academic performance. However, evidence indicates that the relationship between EI and anxiety during examinations is complex [9]. Those who are better at perceiving, understanding and managing emotions are considered to have better skills in handling emotional distress. Nevertheless it is suggested that sometimes those who are better at emotional perception are more likely to pick up stressful stimuli which in turn could contribute to increased level of anxiety leading to adverse outcomes [9].

There are number of studies from different populations showing that EI is related to academic and professional success in many fields including medicine [10–16]. Individuals with higher EI are perceived to have better interpersonal relationships and considered by their peers to be more affable [17].The positive relationships could affect general intellectual development positively which ultimately leads to higher academic performance [18, 19]. Furthermore the intrapersonal EI which is involved with self-regulation and self-motivation promotes behaviour patterns that improve academic performance [17]. A study done in a group of undergraduate students in USA demonstrated that EI was associated with performance beyond ones general intellectual abilities [20].

Evidence indicate that in clinical practice, EI is related to improved empathy in medical consultation [21], better doctor-patient relationships [21], better clinical performance [22, 23] and higher patient satisfaction [24]. These findings highlight that EI plays a critical role in making a balanced doctor who is competent in practicing both the art and the science of medicine. Moreover the doctors and medical students with higher EI are likely to be more competent with regard to self-care, thus preventing them becoming victims of the inevitable stress associated with the medical profession.

Globally, data regarding the effect of EI on the academic performance of medical undergraduates are limited and there is only one publication from Sri Lanka [8]. Being a relatively new concept may be partly responsible for the lack of data. Absence of a universally accepted method or a gold standard to measure EI may be another contributing factor. There are several tools to measure EI but all of them have their own strengths and weaknesses and no single test is considered to be the gold standard [10].

We studied EI and its effect on academic performance in a sample of medical undergraduates of a Sri Lankan university using Genos EI Inventory which is an emotional ability-based instrument [25]. In this study we decided to focus on the academic performance of students at their final year examination, for two reasons. Firstly the final year is considered to be the most emotionally demanding year for a medical student. Secondly a higher level of EI is more likely to enhance academic performance at the final year examination where assessment of skills related to clinical performance plays a major role and EI is important in adjusting to the clinical work in the hospital environment.

## Methods

### Study setting and participants

This is a cross-sectional study, done at the medical faculty of a selected university in Sri Lanka. In Sri Lanka there are eight state universities that offer undergraduate medical education and one of them was selected purposively for this study. All those who were registered at the selected medical faculty and sat for the final MBBS examination in their first attempt in January 2016 were eligible. They were contacted via email and telephone. Assessment of EI and collection of socio-demographic data were done using electronic forms. Participant Information Sheet and Consent Form were incorporated into the electronic form along with the study instruments. When needed, clarifications related to study participation were made via email and telephone. Those who were consenting could proceed to complete the study instruments. Data collection was done from 1st to 31st May 2016. Participation was purely based on goodwill and no incentives were provided.

### Assessment of emotional intelligence

The study instrument we used to assess the EI was self-administered Genos Emotional Intelligence Full Version (Genos EI inventory) [25]. This tool has 17 items in seven different domains of EI. It quantifies how frequently a person behaves in an emotionally intelligent manner. The seven domains include: emotional self-awareness, emotional expression, emotional awareness of others, emotional reasoning, emotional self-management, emotional management of others and emotional self-control [25]. The items are scored on a five-point Likert scale, consisting of ‘Almost Never’, ‘Seldom’, ‘Sometimes’, ‘Usually’ and ‘Almost Always’. Completion of the tool takes about 20 min.

The maximum score for each item in this 70 item tool is 5. Thus the maximum total score is 350 and maximum score for each domain is 50. Without a wide knowledge of the characteristics of the normative sample, interpretation of scores obtained from a psychometric tool is difficult. Therefore, to make the interpretation easy, it is recommended to convert raw scores to percentile scores when this tool is used for selection of professionals for employment [25]. However when the tool is used for research purposes the recommendation is to analyses the raw scores, as statistical analysis becomes demanding if percentile scores are used [25]. Genos EI inventory focuses upon EI ability. It does not measure the cognitive capacity of an individual in relation to recognition and management of emotions. The Genos EI scores indicate the rate at which a person actually behaves in an emotionally intelligent manner. In other words Genos EI inventory measures a person’s ‘regular EI performance’ and not the ‘best possible EI performance’ [25].

The age range of the normative sample utilised to develop Genos EI inventory was 18 to 76 years [25]. Therefore, it is considered applicable to adults over 18 years of age. The scores obtained from Genos EI inventory are valid and reliable. Theses scores, were shown to have high levels of internal consistency reliability (0.90 at the total scale level and 0.70 at the subscale level) and test-retest reliability (total score correlations of 0.83 at 2 months and 0.72 at 8 months) [25]. Further, there is ample evidence to support an appreciable amount of validity across several dimensions including factorial, concurrent, predictive and discriminant validity [25]. Administration of this tool to individuals of several countries (including Asian countries) who speak English has shown that it produces high quality data [25]. In our study Genos EI inventory was administered to the study participants in English. The participants’ study medium over the 5 years spent in the medical faculty was English and thus the language was not a limiting factor. However, this tool is designed for assessment of emotional intelligence at workplaces and therefore a glossary was provided to explain how the words like “work”, “organization”, “stakeholders” and “colleagues” should be interpreted in the study setting. Prior to the commencement of study, written permission was obtained from the publishers of Genos EI Inventory to use it for research purposes.

### Socio-demographic data

The second part of the electronic form used for data collection consisted of variables postulated to be important for EI and/or academic performance. The variables included: gender, age, place of residence during university years, monthly income of the family, number of siblings, losing a parent at age < 18 years, father’s and mother’s level of education, and nature and extent of involvement in extracurricular activities in the school / university. Questions were included to assess self-perceived relationship with the father and the mother and the responses were recorded on a four-point Likert scale that included the responses “very good”, “good”, “average” and “poor”. The self-perceived level of family support was recorded on a five-point Likert scale from “very good” to “very poor”. Further questions examined whether the participant is socializing well in the university environment as self-perceived, whether the participant is a religious person as self-perceived, whether the participant enjoys studying medicine and whether the participant is satisfied with the facilities available for learning. The responses to these questions were recorded on a five-point Likert scale from “strongly agree” to “strongly disagree”. The questionnaire was administered in English and was pilot tested before administering to the study participants.

### Assessment of academic performance

Academic performance of the study participants was assessed with the overall result at the final MBBS examination. The results were obtained from the records maintained by the Examination Unit of the respective medical faculty. Research team did not have access to raw marks due to confidentiality issues. The participants were categorized into 4 results-groups: second-class upper division, second-class lower division, pass and repeat. Those who have passed all the final year subjects but have not obtained a class were categorized as “pass” and those who have not passed one or more subjects were categorized as “repeat”. In the study sample there were no students who obtained a first class.

In the university where the study was carried out, there are six subjects for the final MBBS examination: Medicine, Surgery, Paediatrics, Obstetrics and Gynaecology, Psychiatry and Family Medicine. Assessment of each subject comprises written assessments (multiple choice questions and structured essay questions) and clinical examinations. Twenty percent (20%) of the final MBBS marks for each subject come from the continuous assessments comprising Objective Structured Clinical Examinations (OSCEs) and log book assessments. Marks for individual subjects are expressed as percentages. Whether a student gets a pass in the individual subject is based on this percentage mark and the pre-defined examination by-laws in the university. Candidates who pass all the subjects at the first attempt fulfilling the pre-defined criteria in the examination by-laws are eligible for classes. The average of percentage marks for all six subjects is considered for granting classes.

### Statistical analyses

Data were analysed using SPSS version 23. Percentages and means were used to describe socio-demographic factors, academic performance and EI of the study participants. Associations between categorical data were assessed with Chi-square test and Fisher’s Exact test as appropriate. To compare means between groups, independent-sample t-test was used when there were only two groups and ANOVA was used when there were more than two groups. Multiple linear regression analysis was done to find the socio-demographic factors that independently predicted the EI score. Ordinal regression analysis, was done to determine the independent predictors of final MBBS results category when all four categories (repeat, pass, second-class lower division, second-class upper division) were considered. A *p* value of less than 0.05 was considered as statistically significant.

For the analytical purposes, the socio-demographic factors were dichotomised and coded as “yes” or “no” as follows: residency at home during all 5 years of the university life, residency at hostel / boarding house during all 5 years of university life, family income ≥ Rs.100,000/= per month, family income ≥ Rs.50,000/= per month, having siblings (one or more), level of education of mother is up to Advanced Level (A/L) examination or above, level of education of father is up to A/L examination or above, losing a parent at age < 18 years, aesthetic activities in school and university (engaged in music, dancing, art, drama, literature or photography for > 1 h per week), sports in school and university (engaged in indoor / outdoor, individual / team sports for > 1 h per week), good family support (good and very good ratings), good relationship with the father and mother ((good and very good ratings), socializing well in the university environment (agree and strongly agree ratings), religious person (agree and strongly agree rating), enjoys studying medicine (agree and strongly agree ratings), satisfied with the facilities available for learning (agree and strongly agree ratings).

### Ethical aspects

Ethics Review Committee of the Faculty of Medical Sciences, University of Sri Jayewardenepura, Sri Lanka granted approval for this study. [Ref. No.20/16]. Study participants gave informed consent prior to recruitment.

## Results

Of 148 eligible subjects, 130 responded resulting in a response rate of 88%. The mean age was 26.3 ± 1 years and 61.5% were females. The gender distribution was similar to that of the entire study population consisting of all eligible subjects (females – 61.5%). When the distribution of results categories is considered, the percentage of repeat students was slightly lower (17% vs 20%) and the percentage of students with classes was slightly higher (51% vs 47%) among those who participated in the study as compared to the entire study population. The socio-demographic factors of the study participants are detailed in Table 1.

**Table 1:** Socio-demographic factors of the study participants (*n* = 130)

Socio-demographic factor	n (%)
Gender	
Female	80 (61.5)
Male	50 (38.5)
Residence – home in all 5 years	
Yes	29 (22.3)
No	101 (77.7)
Residence – hostel / boarding house in all 5 years	
Yes	43 (33.1)
No	87 (66.9)
Monthly income of the family (Sri Lankan Rupees)	
≥ 100,000/=	35 (26.9)
50,000/= to 100,000/=	65 (50.0)
< 50,000/=	30 (23.1)
Having siblings	
Yes	121(93.1)
No	9 (6.9)
Lost a parent at age < 18 years	
Yes	9 (6.9)
No	121 (93.1)
Level of education of mother	
below O/L	4 (3.1)
upto O/L	23 (17.7)
upto A/L	58 (44.6)
above A/L	45 (34.6)
Level of education of father	
below O/L	3 (2.3)
upto O/L	17 (13.1)
upto A/L	51 (39.2)
above A/L	59 (45.4)
Relationship with mother	
Very good	99 (76.2)
Good	27 (20.8)
Average	4 (3.1)
Poor	0 (0)
Relationship with father	
Very good	68 (52.3)
Good	43 (33.1)
Average	17 (13.1)
Poor	2 (1.5)
Level of family support	
Very good / Good	126 (96.9)
Not sure	0 (0)
Very poor / Poor	4 (3.1)
Aesthetic activities in school	
Yes	113 (86.9)
No	17 (13.1)
Aesthetic activities in university	
Yes	73 (56.2)
No	57 (43.8)
Sports in school	
Yes	83 (63.8)
No	47 (36.2)
Sports in university	
Yes	27 (20.8)
No	103 (79.2)
Socialize well in the university	
Strongly agree / Agree	98 (75.4)
Not sure	22 (16.9)
Strongly disagree / Disagree	10 (7.7)
Religious person	
Strongly agree / Agree	95 (73.1)
Not sure	21 (16.2)
Strongly disagree / Disagree	14 (10.7)
Enjoy studying medicine	
Strongly agree / Agree	79 (60.8)
Not sure	29 (22.3)
Strongly disagree / Disagree	22 (16.9)
Satisfied with facilities available for learning	
Strongly agree / Agree	85 (65.4)
Not sure	26 (20.0)
Strongly disagree / Disagree	19 (14.6)

*O/L* Ordinary Level, *A/L* Advanced Level

### EI score

Mean total EI score of the study sample was 241.5 ± 25.9. Total EI score was higher among females (245.5 vs 235.1; *p* = 0.045), those with good family support (243.2 vs 186.8; *p* < 0.001), those who were involved in aesthetic activities in the school (243.7 vs 226.9; *p* = 0.013) and in the university (246. 8 vs 234.6; *p* = 0.010), those who socialized well in university (246.7 vs 225.1; *p* < 0.001), those who enjoyed studying medicine (246.7 vs 233.4; *p* = 0.007) and those who were satisfied with facilities available for learning (247.5 vs 230.1; *p* < 0.001). Residence during university years, family income, having siblings, losing a parent at age < 18 years, education level of parents or being a religious person didn’t have a significant association with EI (Table 2).

**Table 2:** Socio-demographic factors and total EI score (*n* = 130)

Socio-demographic factor	Total EI score	*P* value*
Gender		
Female	245.5	*0.045* ^†^
Male	235.1	
Residence – home in all 5 years		
Yes	244.5	0.481
No	240.6	
Residence – hostel / boarding house in all 5 years		
Yes	239.4	0.527
No	242.5	
Monthly income of the family (Sri Lankan Rupees)		
≥ 100,000/=	242.3	0.830
< 100,000/=	241.2	
Monthly income of the family (Sri Lankan Rupees)		
≥ 50,000/=	242.5	0.550
< 50,000/=	238.7	
Having siblings		
Yes	241.4	0.866
No	242.9	
Lost a parent at age < 18 years		
Yes	241.7	0.982
No	241.5	
Level of education of mother – upto A/L or above		
Yes	243.3	0.240
No	234.4	
Level of education of father – upto A/L or above		
Yes	245.7	0.087
No	237.8	
Good relationship with mother		
Yes	242.8	0.134
No	199.8	
Good relationship with father		
Yes	244.0	0.054
No	225.6	
Good family support		
Yes	243.2	*< 0.001* ^†^
No	186.8	
Aesthetic activities in school		
Yes	243.7	*0.013* ^†^
No	226.9	
Aesthetic activities in university		
Yes	246.8	*0.010* ^†^
No	234.6	
Sports in school		
Yes	243.8	0.168
No	237.3	
Sports in university		
Yes	236.8	0.296
No	242.7	
Socialize well in the university		
Yes	246.7	*< 0.001* ^†^
No	225.1	
Religious person		
Yes	244.0	0.117
No	234.7	
Enjoy studying medicine		
Yes	246.7	*0.007* ^†^
No	233.4	
Satisfied with facilities available for learning		
Yes	247.5	*< 0.001* ^†^
No	230.1	

*EI* Emotional Intelligence, *A/L* Advanced Level

^*^ *P* value based on student t-test; ^†^ *p* value < 0.05

Among different domains, mean score was highest for Emotional Self-Awareness (36.8/50) and lowest for Emotional Expression (32.6/50). Females had higher scores across all domains and the differences were significant for Emotional Self-Awareness (*p* = 0.033) and Emotional Reasoning (*p* = 0.041) (Table 3).

**Table 3:** Emotional intelligence and academic performance of the study participants

	All (*n* = 130)	Male (*n* = 50)	Female (*n* = 80)	*P* value
EI Scores				
Mean total EI Score (out of 350)	241.5	245.5	235.1	*0.045* ^#†^
Mean scores for EI domains (out of 50)				
Emotional Self-Awareness	36.8	35.5	37.6	*0.033* ^#†^
Emotional Expression	32.6	31.8	33.2	0.069^#^
Emotional Awareness of Others	35.4	34.5	35.9	0.090^#^
Emotional Reasoning	34.6	33.5	35.2	*0.041* ^#†^
Emotional Self-Management	33.3	32.4	33.9	0.119^#^
Emotional Management of Others	35.5	35.1	35.7	0.454^#^
Emotional Self-Control	33.4	32.3	34.1	0.073^#^
Final MBBS results (%)				
Second-class upper division	8.5	4.0	11.3	*0.009* ^##†^
Second-class lower division	43.1	34.0	48.8	
Pass	31.5	32.0	31.3	
Repeat	16.9	30.0	8.8	

*EI* Emotional Intelligence

^#^ *P* value based on student t-test; ^##^ *P* value based on chi-square test; ^†^ *p* value < 0.05

Multiple linear regression analysis indicated that having good family support (*p* = 0.002), socializing well in university (*p* = 0.024) and being satisfied with facilities available for learning (*p* = 0.002), were independent predictors of EI (Table 4).

**Table 4:** Multiple linear regression for total EI score with socio-demographic factors (*n* = 130)

Model	Unstandardized Coefficients		Standardized Coefficients	t	Sig	95.0% Confidence Interval for B	
	B^*^	Std. Error	Beta			Lower Bound	Upper Bound
1 (Constant)	161.759	12.307		13.144	000	137.390	186.129
Female gender	5.735	4.146	108	1.383	.169	−2.474	13.945
Good family support	36.499	11.585	.244	3.151	*.002* ^*†*^	13.559	59.439
Aesthetic activities in school	6.947	6.232	.091	1.115	.267	−5.393	19.286
Aesthetic activities in University	−.451	4.565	−.009	−.099	.922	−9.489	8.588
Socialize well in the university	10.626	4.662	.177	2.279	*.024* ^*†*^	1.394	19.858
Enjoy studying medicine	4.916	4.047	.093	1.215	.227	−3.097	12.928
Satisfied with facilities for learning	13.029	4.012	.240	3.247	*.002* ^*†*^	5.084	20.973

^*^Multiple Linear Regression used Enter Method; ^†^ *p* value < 0.05; *EI* Emotional Intelligence

### Academic performance

At the final MBBS examination 51.6% obtained classes, 31.5% passed the examination without classes and 16. 9% got repeated. Females have performed better than males at the final MBBS examination (*p* = 0.009). The classes were more among females and the repeat rate was higher among males (Table 3). On ordinal regression analysis, which considered all four results categories (repeat, pass, second-class lower division and second-class upper division) as outcomes, female gender in contrast to male gender predicted a higher grade at the final MBBS examination (β - 0.874 (95% CI 0.191-1.556); *p* = 0.012) (Table 5).

**Table 5:** Ordinal regression analysis for final MBBS results categories with total EI score and gender (*n* = 130)

	Estimate	Std. Error	Wald	df	Sig.	95% Confidence Interval	
						Lower Bound	Upper Bound
Threshold							
[finalmb_R = 1]	3.244	1.577	4.232	1	.040	.153	6.335
[finalmb_R = 2]	4.938	1.613	9.366	1	.002	1.776	8.100
[finalmb_R = 3]	7.526	1.677	20.129	1	.000	4.238	10.814
Location							
Total EI	*.018*	.007	7.547	1	*.006* ^†^	.005	.031
Female gender	*.874*	.348	6.286	1	*.012* ^†^	.191	1.556

^†^ *p* value < 0.05; *EI* Emotional Intelligence

When the two broad categories of repeat and non-repeat (comprising the categories: pass, second-class lower division and second-class upper division) groups were considered***,*** examination performance was better among participants whose mother’s education was up to A/L or above (88.3% vs 63%; *p* = 0.004) and father’s education was up to A/L or above (91.5% vs 77.1%; *p* = 0.027) [see Additional file 1]. Those who enjoyed studying medicine showed a higher non-repeat rate than those who did not (89.3% vs 72.5%; *p* = 0.010). Non-repeat rate was also higher among those reported to have good family support (84.9% vs 25%; *p* = 0.015).

### Relationship between EI and academic performance

One way ANOVA showed that the total EI score was higher among those with better academic performance. The Mean total EI scores among those in categories second-class upper division, second-class lower division, pass and repeat were 249.4, 246.6, 240.2 and 227, respectively (*p* = 0.015). Ordinal regression analysis indicated that, after adjusting for gender, total EI was an independent predictor of academic performance at final MBBS examination (β - 0.018 (95% CI 0.005-0.031); *p* = 0.006) (Table 5).

## Discussion

Our study shows that EI is an independent predictor of academic performance at final MBBS examination amongst medical undergraduates of a selected university in Sri Lanka. There is paucity of data related to the relationship between EI and academic performance in university students in Sri Lanka.

Despite the absence of incentivization, we obtained a high response rate which is close to 90%. This is a major strength of the study. The study was done at a time when the students were relatively free from other commitments as there was a gap between graduation and internship. We believe that it helped to achieve a high response rate. Even though several tools have been developed to assess EI there is no single test which stands as the gold standard [10]. We assessed the EI with a tool which assesses the EI ability focusing on an individual’s typical EI performance in contrast to the individual’s optimal EI performance. The typical EI performance provides better insight regarding a student’s behaviour in real life settings. This is another strength of our study. We looked at the final MBBS examination results as our primary study outcome because it is considered to be the most important summative assessment faced by the medical undergraduates. Whether a student to be conferred the medical degree or not and the students’ merit order for obtaining the internship appointments are based on the final MBBS examination result. Furthermore the role played by the EI is likely to be more intense at the final MBBS examination where clinical skills are assessed.

Our study findings highlight the important link between the EI and academic success of medical students. There is only one published study from Sri Lanka on the subject and it has found an association between higher EI and better performance at examinations [8]. The findings of our study done in students from a different university strengthen the findings of the previous study while emphasizing the fact that the link between EI and academic performance exists despite different learning environments.

There could be several reasons for the better academic performance among those with higher EI. Those with higher EI could get adjusted to the emotionally demanding medical curriculum more easily and thus perform better. Ranasinghe et al. reported that among medical undergraduates from a different university in Sri Lanka, self-perceived stress score was lower in those with higher EI [8]. Joseph et al. has reported that EI positively predicts performance in emotionally demanding situations [26]. When the different domains of EI are considered, our study sample showed highest scores in the domain “emotional self-awareness”. This is an important attribute in getting adjusted to emotionally demanding environments. The interpersonal EI is also a very useful attribute for a medical student specially, during the final year where bulk of the learning and assessment is dependent on interpersonal relationship with patients. Interpersonal EI is also important for nurturing positive relationships with the doctors and the other healthcare workers in the hospital, which in turn facilitates a smooth learning process. In the study sample the second and third highest scores were observed in the domains “emotional management of others” and “emotional awareness of others”. Both these domains are important for interpersonal EI.

Our findings with regard to the relationship between EI and academic performance are in keeping with the findings of several previous studies from different countries including USA, India and Malaysia [11–13, 20]. Chew et al. reported that in a group of medical students from Malaysia, the total EI score was indicative of how a student performs in the final year examinations but not in the first year examinations [11]. The study done by Ranasinghe et al. has demonstrated the same trend [8].There was an association between higher EI and better academic performance in the final year students but no such relationship was observed in 2nd and 4th year students. A study from United Kingdom also did not show a strong link between EI and academic performance in first-year medical students [27]. A study from USA done in 2nd, 3rd and 5th year students has shown that EI was significantly associated with performance at problem-based learning sessions [16]. However this study failed to show a relationship between EI and year end examinations. A study from Australia has reported that EI is a significant predictor of academic achievement in nursing students [15].

The mean total EI score of our study sample was 69% (241.5 out of 350) of the maximum possible score. However, there is no cutoff level for the raw EI scores to categories the participants as having satisfactory and unsatisfactory EI. The evidence indicate that higher the score better the academic and professional performance [10–16, 22, 23]. The recommendation of the developers of the tool is to convert raw scores to percentile scores and rank the individuals when it is used for recruitment of professionals but in research settings it is not appropriate [25]. Comparison of the raw scores between different studies is difficult as different studies have used different tools to assess the EI. Nevertheless, we found one study previously done in Sri Lanka in Advanced Level students where the same tool (i.e. Genos EI Inventory) was used to assess EI. It reported a higher total EI score among those school children than what we observed among medical students [28]. One would expect EI to improve with age due to influence of experiences in life and maturity but this observation contradicts this perception. In the same study in school children, subgroup analysis indicated that mean EI score was higher among the students in the Science stream than in those in Commerce and Arts streams. Thus it would be interesting to further study whether the Advanced Level Science Stream students who are emotionally more intelligent do not get selected to the medical faculties in Sri Lanka according to the current selection criteria. Currently the only criterion for admission to medical faculties in Sri Lanka is the academic performance at the Advanced Level examination. Imran et al. has reported that medical students in Pakistan have EI scores lower than average people [29].

When we look at the scores obtained in different domains of EI the highest scores were for Emotional Self-Awareness and the lowest scores were for Emotional Expression. This is an important finding because even if one is aware of his/her own emotions if they are not appropriately expressed it is likely to have negative effects such as stress and anxiety which in turn can affect the academic performance. Poor expression of the emotions may be a cultural issue more specific to Asian counties. Literature survey did not reveal any studies exploring this aspect. Nevertheless this finding highlights the importance of giving special attention to improve emotional expression in settings similar to our study setting.

In our study, both academic performance and EI score were higher among females. However ordinal regression analysis indicated that EI score predicts academic performance independent of gender. Gender based differences in EI are not uniform across the studies from different geographical areas in the world. Studies from UK, Ireland and India have shown that females have higher EI scores [13, 27, 30].

The recently published study in Sri Lankan medical students from a different university also showed that EI was higher among female students [8]. However, the previous Sri Lankan study, in school children reported that boys had higher EI score than girls [28]. A study from Australia reported a higher EI score among male medical students [31] and a study from Pakistan reported that there was no gender based difference in EI [29]. Arora et al. did a systematic review and reported that majority of the studies have found that females have higher EI than males [21].

Identifying and understanding the factors influencing EI is important for planning and implementing programmes to improve EI among the students. In addition to the female gender several other factors were found to be associated with higher EI among the study participants. Those who reported to have good family support, those who claimed to be socializing well, those who were satisfied with available facilities, those who enjoyed studying medicine and those who were engaged in aesthetic activities had significantly higher EI scores. However, the relationship between EI and factors like socialization and being satisfied with the available facilities is likely to be a mutually-reinforcing relationship. Regarding the engagement in extracurricular activities, the EI was significantly higher among those involved in aesthetic activities than those who did not but no significant difference was observed among those who did and did not participate in sports activities. A study done in medical students in Singapore has found that those who spent more time on art had higher empathy scores [32]. Despite the common belief, factors such as not having siblings, family income and religiosity did not have a significant effect on the EI. Literature review revealed comparable results observed in other settings. Kumar et al. has reported that meeting friends, physical exercise and recreational activities, were significantly associated with EI in a group of final-year dental students from India. Studies which have evaluated EI in medical students from different years of study, including the study from Sri Lanka, have found that EI scores were generally similar among first and final year students [8, 11, 29].

### Limitations

Firstly, this study was done in a group of students from a single selected university and multicentre studies are needed before arriving at firm conclusions applicable in a generalised manner. The second limitation is that some of the eligible participants refrained from participating. Even though we had a very high response rate close to 90% and the gender distribution in the sample was identical to that of all eligible students, there was a slight discrepancy with regard to the distribution of final MBBS examination result categories. The percentage of students who got repeated was slightly higher when all eligible students were considered. This is likely to be a reflection of low motivation among those with poor academic performance. This selection bias is difficult to avoid in this type of studies. Finally the exam performance and assessment of EI did not happen simultaneously. There was a 3 month gap. Thus the EI results we obtained might not be reflective of the EI of the participants at the time of the exam performance. However the time gap is minimal and previous research indicate that even during the 5 year training in the medical school EI changes minimally [8, 11, 29]. Furthermore the instrument we used to measure EI focuses on the regular or usual EI related behaviour of a person as opposed to the EI at a single point which further mitigates any potential effects of the 3 month gap.

## Conclusions

Among the medical undergraduates of the selected Sri Lankan university, both emotional intelligence and academic performance at the final MBBS examination were higher among females. Independent of gender, academic performance was better in those who were more emotionally intelligent. Several psychosocial factors were found to be independent predictors of emotional intelligence in the study population. The preliminary data from this study suggest that emotional skills development might enhance academic performance of medical undergraduates in Sri Lanka. Further research is needed in this under-explored area.

## Additional file

Additional file 1: Table S1. Socio-demographic factors and academic performance at final MBBS examination (*n* = 130). This table shows the association between socio-demographic factors and examination results at the final MBBS examination. (DOCX 17 kb)

## Abbreviations

A/L: Advanced Level
ANOVA: Analysis of variance
EI: Emotional Intelligence
MBBS: Bachelor of Medicine, Bachelor of Surgery
O/L: Ordinary Level
SPSS: Statistical Package for the Social Sciences
USA: United States of America
