# Supplementary material for: Emotional intelligence and academic performance of medical undergraduates: a cross-sectional study in a selected university in Sri Lanka
Source: BMC Med Educ. 2017 Sep 25;17:176. doi: 10.1186/s12909-017-1018-9 (PMC5613354; doi:10.1186/s12909-017-1018-9)
Supplement: Additional file 1: Table S1. — Socio-demographic factors and academic performance at final MBBS examination (n = 130). This table shows the association between socio-demographic factors and examination results at the final MBBS examination. (DOCX 17 kb) [file 12909_2017_1018_MOESM1_ESM.docx]

**Additional file 1**

**Table S1: Socio-demographic factors and academic performance at final MBBS examination (n=130)**

| **Socio-demographic factor** | **Non-repeat***  **%** | **P value** |
| --- | --- | --- |
| **Gender**  Female  Male | 91.2  70.0 | **0.002^#^** |
| **Residence – home in all 5 years** Yes  No | 93.1  80.2 | 0.158^##^ |
| **Residence – hostel / boarding house in all 5 years** Yes  No | 76.7  86.2 | 0.176^#^ |
| **Monthly income of the family (Sri Lankan Rupees)** ≥100000/=  <100000/= | 91.4  80.0 | 0.123^#^ |
| **Monthly income of the family (Sri Lankan Rupees)** ≥50000/=  <50000/= | 85.4  76.5 | 0.232^#^ |
| **Having siblings**  Yes  No | 83.5  77.8 | 0.648^##^ |
| **Lost a parent at age < 18 years** Yes  No | 82.6  88.9 | 1.000^##^ |
| **Level of education of mother – upto A/L or above** Yes  No | 88.3  63.0 | **0.004^##^** |
| **Level of education of father – upto A/L or above** Yes  No | 91.5  77.1 | **0.027^#^** |
| **Relationship with mother** Good  Not good | 84.1  50.0 | 0.133^##^ |
| **Relationship with father** Good  Not good | 84.8  72.2 | 0.188^##^ |
| **Level of family support** Good  Not good | 84.9  25.0 | **0.015^##^** |
| **Aesthetic activities in school** Yes  No | 84.1  76.5 | 0.436^#^ |
| **Aesthetic activities in university** Yes  No | 86.3  78.9 | 0.267^#^ |
| **Sports in school** Yes  No | 79.5  89.4 | 0.150^#^ |
| **Sports in university**  Yes  No | 70.4  86.4 | 0.079^##^ |
| **Socialize well in the university** Yes  No | 84.7  78.1 | 0.390^#^ |
| **Religious person** Yes  No | 84.2  80.0 | 0.570^#^ |
| **Enjoy studying medicine** Yes  No | 89.9  72.5 | **0.010^#^** |
| **Satisfied with facilities available for learning** Yes  No | 84.7  80.0 | 0.496^#^ |

*Non-repeat group comprises the three results categories: pass, second-class lower division and second-class upper division

^#^P value based on Chi-square test; ^##^ P value based on Fisher’s exact test

EI – Emotional Intelligence; A/L – Advanced Level;
